# Non-Traditional Aspects of Renal Diets: Focus on Fiber, Alkali and Vitamin K1 Intake

**DOI:** 10.3390/nu9050444

**Published:** 2017-04-29

**Authors:** Adamasco Cupisti, Claudia D’Alessandro, Loreto Gesualdo, Carmela Cosola, Maurizio Gallieni, Maria Francesca Egidi, Maria Fusaro

**Affiliations:** 1 Department of Clinical and Experimental Medicine, University of Pisa, Pisa 56126, Italy; dalessandroclaudia@gmail.com (C.D.); m.egidi@ao-pisa.toscana.it (M.F.E.); 2 Department of Emergency and Organ Transplantation—Nephrology, Dialysis and Transplantation Unit, University of Bari Aldo Moro, Bari 70124, Italy; loreto.gesualdo@uniba.it (L.G.); carmela.cosola@uniba.it (C.C.); 3 San Carlo Borromeo Hospital, ASST Santi Paolo e Carlo, University of Milano, Milano 20153, Italy; maurizio.gallieni@unimi.it; 4 National Research Council (CNR), Institute of Clinical Physiology (IFC), Pisa and Department of Medicine, University of Padua, Padua 35122, Italy; dante.lucia@libero.it

**Keywords:** Renal diets, Vitamin K1, PRAL, fiber, gut microbiota, uremic toxins, low protein diet, renal nutrition, metabolic acidosis, CKD

## Abstract

Renal diets for advanced chronic kidney disease (CKD) are structured to achieve a lower protein, phosphate and sodium intake, while supplying adequate energy. The aim of this nutritional intervention is to prevent or correct signs, symptoms and complications of renal insufficiency, delaying the start of dialysis and preserving nutritional status. This paper focuses on three additional aspects of renal diets that can play an important role in the management of CKD patients: the vitamin K1 and fiber content, and the alkalizing potential. We examined the energy and nutrients composition of four types of renal diets according to their protein content: normal diet (ND, 0.8 g protein/kg body weight (bw)), low protein diet (LPD, 0.6 g protein/kg bw), vegan diet (VD, 0.7 g protein/kg bw), very low protein diet (VLPD, 0.3 g protein/kg bw). Fiber content is much higher in the VD and in the VLPD than in the ND or LPD. Vitamin K1 content seems to follow the same trend, but vitamin K2 content, which could not be investigated, might have a different pattern. The net endogenous acid production (NEAP) value decreases from the ND and LPD to the vegetarian diets, namely VD and VLPD; the same finding occurred for the potential renal acid load (PRAL). In conclusion, renal diets may provide additional benefits, and this is the case of vegetarian diets. Namely, VD and VLPD also provide high amounts of fibers and Vitamin K1, with a very low acid load. These features may have favorable effects on Vitamin K1 status, intestinal microbiota and acid-base balance. Hence, we can speculate as to the potential beneficial effects on vascular calcification and bone disease, on protein metabolism, on colonic environment and circulating levels of microbial-derived uremic toxins. In the case of vegetarian diets, attention must be paid to serum potassium levels.

## 1. Introduction

Nutritional treatment plays a pivotal role in the care of patients affected by chronic kidney disease (CKD). Dietary interventions aim to reduce the burden of uremic toxins in order to prevent or correct signs, symptoms and complications of chronic renal insufficiency, thereby delaying the need for dialysis [1,2]. 

Healthy dietary patterns, including DASH (Dietary Approaches to Stop Hypertension) diet and Mediterranean diets, may be useful for CKD prevention or when restriction of phosphate or nitrogen load are not necessary or feasible [3,4,5]. In the early phases of CKD, normalization of protein (0.8 g/kg/day) and salt (6 g/day) intake are usually implemented with the aim of enhancing the anti-proteinuric response to renin-angiotensin-aldosterone-system (RAAS) inhibitors, and to prevent abnormalities of calcium-phosphate metabolism [6]. 

When renal insufficiency progresses, more specific renal diets are prescribed to reduce uremic toxin retention, phosphate and sodium load. They include low-protein regimens, namely the conventional, animal-based low protein (0.6 g/kg/day), or the vegan low protein (0.7 g/Kg/day) diet, or the vegan, very low protein (0.3 g/kg/day) diet supplemented with essential amino acids and keto-acids [1,7]. 

These renal diets are based on the control of protein, sodium and phosphate intake, while preserving nutritional status by high energy intake and adequate essential amino acids supply. 

Hence, a progressive protein restriction is highly recommended along with the progression of renal insufficiency [8,9]; however, this is only one aspect of the dietary management of CKD patients. Additional features, which are strictly related to protein intake, include changes in sodium, phosphorus and energy intake, as well as in the source of protein and lipids, namely of animal or plant origin. A major point is that energy intake must cover the energy requirement (30–35 Kcal/Kg/day), to allow a correct metabolic adaptation in the course of protein-restricted regimens, thus preventing negative nitrogen balance [10] and protein-energy wasting. 

Apart from these traditional dietary manipulations, dietary supplements may play an important role in obtaining both beneficial effects and nutritional safety in the CKD patient [11,12]. Essential amino acids plus ketoacids supplementation is mandatory in the course of a very low-protein diet in order to assure an adequate essential amino acid supply: the goal is to obtain the beneficial effect of a severe protein restriction while preventing malnutrition [13,14]. Calcium, iron, Polyunsaturated Fatty acids (PUFAs) and native Vitamin D represent other types of supplements potentially useful in CKD patients [11].

However, due to novel evidence in the physiopathology of CKD and its complications, other non-traditional dietary aspects should be considered in the nutritional management of CKD patients. This is the case of Vitamin K, fibers and alkali intake. 

Vitamin K has a role in the carboxylation of matrix γ-carbossiglutammate (GLA) protein (MGP) and bone GLA protein (BGP or osteocalcin). Consequently, vitamin K deficiency is associated with an increased risk of vascular calcifications and bone demineralization [15]. The relative importance of vitamin K1 (phylloquinone) and K2 (menaquinones) vitamers is still under active investigation [16].

Fiber intake has a major role in the modulation of intestinal microbiota metabolism towards saccharolytic direction and in lowering proteolytic-derived uremic toxins [17,18,19,20]. Moreover, dietary fibers increase bowel transit. In contrast, constipation, or reduced bowel transit time, worsens microbiota dysbiosis and contributes to the uremic status and to electrolyte imbalance, namely hyperkalemia [21]. 

Reduced dietary acid load is crucial to prevent or correct metabolic acidosis, which plays a major role in causing protein and amino acid catabolism, along with insulin resistance. Therefore, metabolic acidosis leads to maladaptation to protein restriction, as well as electrolyte disturbances and bone damage. In CKD patients, correction of metabolic acidosis may even be important to delay the progression of renal damage [22]. 

This paper analyzes the content of Vitamin K1, alkali and fiber of different renal diets used in pre-dialysis CKD patients, and deals with the importance of these nutrients within the dietary management of CKD. 

## 2. Materials and Methods

We examined the energy and nutrients composition of four types of renal diets [23] according to their protein content: normal diet (ND, 0.8 g protein/kg body weight), low protein diet (LPD, 0.6 g protein/kg bw): vegan diet (VD, 0.7 g protein/kg bw), very low protein diet (VLPD, 0.3 g protein/kg bw).

For each type of diet, 12 daily meal plans (adapted for a 70 Kg patient) were analyzed and the results reported as daily average values. The meal plans are provided to the patients in leaflet form during the nutritional counseling.

Daily meal plans were prepared according to the principles summarized in Table 1, and foods were selected to ensure an adequate variety across days. Table 1 also shows the main characteristics of the studied renal diets, according to quantity and source of protein content.

For each of the four diets, we estimated the energy and nutrient intake, namely protein, carbohydrates, lipids, unsaturated fatty acids, saturated fats, cholesterol, calcium, phosphorus, magnesium, iron, zinc, copper, fiber, and vitamins A, C, B1, B2, B3, K1, D. Composition data were obtained using the food composition Tables of the National Institute of Nutrition and of the European Institute of Oncology Database Edition 2008 [24]. Vitamin K1 content was derived from the “United States Department of Agriculture (USDA) National Nutrient Database for Standard Reference”, referring to over 900 foods including foods typical of the Mediterranean diet [25]. Vitamin K2 intake could not be adequately estimated for lack of reference tables and for the difficulty in determining its intestinal synthesis by the microbiota.

The daily energy and nutrients intake were reported as average of the 12 day meal plans for each type of diet. We also calculated the potential renal acid load (PRAL) and the net endogenous acid production (NEAP). These parameters represent the non-volatile acid load derived from the diet, estimated by the production of non-volatile acids and bases produced during digestion based on known nutritional content.

PRAL was calculated by the following formula as described by Remer and Manz [26]: 

PRAL (mEq/day) = (0.49 × protein, g/day) + (0.037 × phosphorus, mmol/day) − (0.021 × potassium, mmol/day) − (0.026 × magnesium, mg/day) − (0.013 × calcium, mg/day). 

NEAP was calculated by the formula described by Frassetto et al. [27].

NEAP(mEq/day) = (54.5 × protein (g/day)/potassium, (mmol/day)) − 10.2.

### Statistical Analysis

Statistical analysis was performed using SPSS 19.0 (SPSS Inc, Illinois, USA) the company, the city, the country) for Windows. Data are expressed as mean ± standard deviation or as median and interquartile range when appropriate.

Intra- and intergroup statistical comparisons of nutrients and energy content of the four diets were performed using one-way Analysis of Variance (ANOVA) and the Bonferroni post hoc test. Differences were considered to be statistically significant when *p* < 0.05.

## 3. Results

Table 1 shows the main characteristics of the studied renal diets. 

Table 2 reports the energy and nutrients content of the studied renal diets. Significantly different levels of protein, phosphorus and sodium content are the main features of the diets.

Table 3 shows the fiber, potassium, magnesium and vitamin K1 content of the dietary patterns for CKD patients, normalized per 1000 Kcal. The fiber content is much higher in the VD and in the VLPD than in the Normal diet ND or in the LPD. The vitamin K1 content also seems to follow this trend.

**Figure 1:**
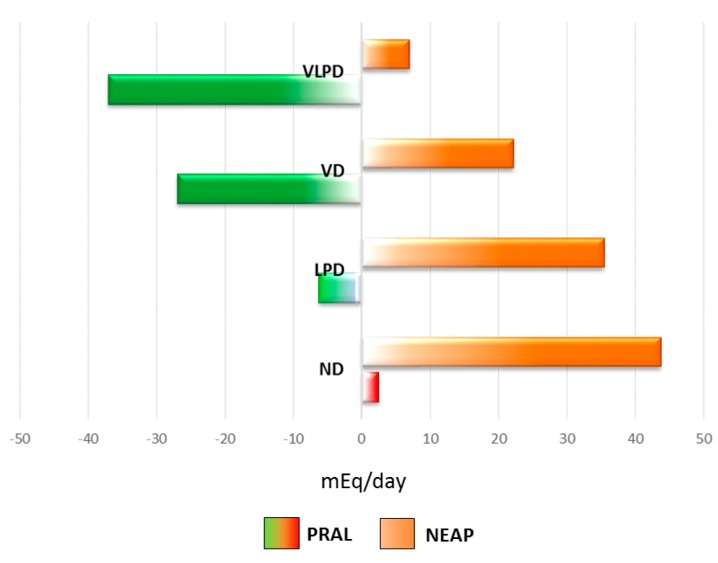
The Potential renal acid load (PRAL) and the net endogenous acid production (NEAP) of the four studied renal diets, expressed as mEq/day. ND: normal diet (0.8 g protein/kg body weight); LPD: low protein diet (0.6 g protein/kg body weight); VD: vegan diet (0.7 g protein/kg body weight); VLPD: very low protein diet (0.3 g protein/kg body weight).

The net endogenous acid production (NEAP) value decreases from the normal diet to the vegetarian diets, namely VD and VLPD (Figure 1).

We calculated the potential renal acid load (PRAL) value to compare the acidic/alkalizing power of the four dietary patterns and, as expected, it decreases from the normal diet to the vegetarian diets (Figure 1).

Regarding the magnesium content, only the VD covers the levels of 240 mg/day recommended for the adult general population, due to legumes and grains that are highly represented in this diet.

## 4. Discussion

The common western dietary habits are excessively rich in protein and low in fruit and vegetables, grains, and fibers, considering the recommendations of the World Health Organization WHO for the general population [28]: 0.83 g of proteins/Kg/day, 6 grams/day of sodium chloride and 700 mg/day of phosphorus. Actually, the usual protein intake is over 1.2 g proteins/kg/day, the salt intake largely overcome 10–12 g/day , and phosphorus is higher than 1200–1700 mg/day. Thus, it is not surprising that prescription of a “normal” diet, that is a diet covering the WHO recommendations, may be a successful first-step intervention able to reduce sodium load, nitrogen waste product retention and calcium-phosphate metabolism abnormalities. 

While adopting healthy dietary patterns, such as the DASH or Mediterranean diets, may be useful as a prevention strategy in a population-based setting, specific renal diets aimed at protein, sodium and phosphorus controlled intake may be needed in advanced stages of renal insufficiency. The aim of nutritional treatment in advanced CKD is to prevent or correct signs, symptoms of chronic renal failure, prevent protein energy wasting (PEW) and delay the start of dialysis by reducing toxin retention, mainly derived by catabolism of dietary protein load. This is the reason why dietary manipulation in renal patients is historically related to protein intake, which is strictly linked to sodium and phosphorus intake. 

This paper focused on additional aspects, namely vitamin K1 and fiber content, and alkalizing power of these renal diets, factors that can play an important role in the management of CKD patients. 

The fiber content is quite high in VD and VLPD (Table 3) and it meets the recommended level of 20–30 g/day for CKD patients [29] both in the VD and in the VLPD (Table 4).

Interestingly, the food industry, in response to increased consumer sensitivity to health topics, is becoming more attentive to fiber content in processed food. Moreover, in the context of protein-free food products specific for CKD patients, in the last year more fibers have been added to regular and even to the protein-free bread or pasta with the aim to reduce the glycemic index, as well as for technological purposes. In particular, the addition of inulin modulates microbiota metabolism and the high fiber intake of vegan diet may have favorable effects on intestinal microbiota. 

A great bulk of literature is highlighting the dynamic link between gut microbiota and the human body pathophysiology, including kidneys and the CKD status [31,32]. Beyond the microbiota activity affecting the whole organism at a systemic level, the gut-kidney axis presents a particularly intimate connection [31].

The microbiota affects human pathophysiology through its metabolites, derived from the saccharolytic (fermenting complex carbohydrates) or the proteolytic (using aminoacids as alternative substrate for energy harvesting) catabolism of food reaching the colon [33]. While the former is believed to be a “beneficial” pathway thanks to the downstream metabolites (mainly Short Chain Fatty Aminoacids—SCFAs) with anti-inflammatory, immune-modulating and gut integrity-promoting action [34], an unbalance towards the latter is noxious because it results in overproduction of many toxic metabolites, such as amines, indoles, phenols, hydrogen sulphide and secondary bile acids [33,35]. For this reason, a “healthy gut” possesses a metabolism that is mainly saccharolytic [33,36]. 

Moreover, uremia impacts the colonic environment exacerbating the dysbiosis status already present in CKD patients, often caused by dietary restrictions in fiber content [37] and altered assimilation of proteins in the intestine, leading to increased availability of colonic amino acids as fermentation substrates [38,39]. In a situation of impaired kidney filtration capacity, the colon acquires a replacement role for the excretion of urea and oxalate: this process alters colonic pH and promotes the overgrowth of potentially pathogenic, proteolytic microbial species, leading to a further increase of uremic toxins production, in a vicious circle [33].

Another pathologic aspect often observed in CKD patients is the “leaky gut” syndrome. Alteration of gut microenvironment, beyond promoting dysbiosis, causes the derangement of colonic epithelial barrier, leading to gut permeability. A leaky gut allows translocation to the circulation of bacteria and microbial fragments, such as bacterial lipopolysaccharides (LPS). This phenomenon is believed to trigger low-grade systemic inflammation, worsening a clinical frame already compromised by uremia [40]. 

The good news is that microbiota metabolism is susceptible of manipulation by dietary matrices. Diets abundant in fiber-rich foods (fruits, vegetables and legumes), such as the Mediterranean or purely vegetarian diets, have the potential to shift the microbial metabolism in saccharolytic direction, with the release of SCFAs and the reduction of uremic toxins [19,20,33]. 

Unfortunately, current dietary management of CKD patients often foresees a strict restriction of most fruits, vegetables and legumes, corresponding to the richest suppliers of dietary fibers. This indication, although justified by the need to control potassium levels, leads to deleterious effects for the colonic health and worsens the microbiota dysbiosis typical of the uremic patient. 

Scientific evidence suggests that this tendency should be reverted. Fiber intake in the dietary management of CKD should be encouraged for its ability to modulate microbiota metabolism in saccharolytic direction and to lower proteolytic-derived uremic toxins [17,18,19,20]. Moreover, fibers increase bowel transit, which is desirable in CKD patients. Reduced bowel transit worsens microbiota dysbiosis and contributes to the uremia status and to electrolyte imbalance [21]. It is meaningful that, in an animal study, even treating the constipation alone with a pharmacologic approach resulted in microbial probiotic species recovery, and circulating microbial toxins reduction [41]. 

Increased fiber intake could also be beneficial for gut permeability. Some studies indicate that SCFAs, saccharolytic metabolites, could be able to promote the epithelial integrity [42], even though this aspect remains to be demonstrated in vivo and is worthy of being investigated in clinical trials.

In the four diets we examined, the NEAP and the PRAL values were significantly lower in vegetarian diets, namely VD and VLPD, than in ND.. The PRAL value of the VD is consistent with what reported in the literature (−26.9 ± 15.4 mEq/day in respect to an average value of −20 mEq/day of a Vegan diet). The PRAL of the VLPD is 37.6% higher than that of the VD. These results were expected, as PRAL depends on protein, phosphate, potassium, calcium and magnesium content of the diet. The PRAL of what we defined a “normal diet” is lower than that reported for a current typical western diet, rich in animal proteins (approximately +50 mEq/day). In fact, plant proteins present in the ND are twice that of the animal proteins and the adequate amount of essential amino acids is guaranteed by the consumption of animal proteins alternate to that of legumes combined to grains, according to the principle of complementarity of proteins. The presence of grains and pulses (together with 4–5 servings of vegetables per day and a phosphate content according to the Recommended Dietary Allowance with the exclusion of cheese) contributes to reduce the PRAL value.

The LPD has a higher amount of animal proteins in comparison to the ND, to counteract the presence of protein-free products and to guarantee an adequate amount of essential amino acids. However, the reduction of total protein intake and phosphate content and the presence of fruit and vegetables contribute to an overall alkalizing effect, even if it is weaker than in vegetarian diets. 

The VD induces a remarkable alkalizing effect due to the exclusive presence of plant foods. Most fruit and vegetables are known to produce alkali when metabolized, contributing to acid neutralization. However, they also contain organic acids (i.e., citric acid) and organic salts as potassium citrate. The organic acids produce hydrogen ions (H^+^) and basic ions when metabolized, while organic salts have a basic ion but no H^+^ so they can buffer H^+^ reducing the acid load. Phosphate content in the VD is higher than that of the LPD but it is prevalently in the form of phytate, thus scarcely absorbable.

The VLPD has the higher negative PRAL value and therefore its alkalizing power is very high: this is due to its low protein content, all from plant origin, and the larger amount of vegetables that contribute to the variety of the diet.

Moreover, these dietary patterns are characterized by a low salt content, as these patients are usually asked by the nephrologist to reduce added salt and to avoid processed foods. This contributes to reduce the acid load, as salt independently increases acid load and reduces serum bicarbonate [43].

A more alkaline diet lowers NEAP and reduces the acid load, contributing to a reduced stimulation of aldosterone, angiotensin II and endothelin, which are involved in dietary acid excretion [22]. In CKD patients a diet rich in fresh fruits and vegetables can control metabolic acidosis similarly to sodium bicarbonate administration [22]. In addition, the VLPD supplemented with EAA and KA reduces metabolic acidosis in patients with advanced CKD [44].

Prevention and correction of metabolic acidosis has paramount importance for limitation of protein and aminoacid catabolism, protection of renal function and reducing the risk of hyperkalemia, hyperphoshatemia and bone mineral loss. 

Thus, VD and VLPD, being vegetarian in nature, have high fiber content, low net acid production and high potassium intake. These are favorable features, but the potassium dietary burden is also relevant concern in CKD patients, and it deserves comments [45]. In patients with preserved renal function and urine volume output, a relevant capacity to excrete potassium by urine still persists. In addition, a significant fecal excretion contributes to maintenance of K balance. Fecal excretion is especially important in dialysis patients, where hyperkalemia seems to be related to both constipation and dietary load [21]. Other relevant causes of hyperkalemia are drugs and acidosis, favoring the shift of potassium from intracellular to extracellular fluids.

Attention must be paid to serum potassium levels in patients on vegetarian diets. Although high dietary intake is not the main pathogenic factor of hyperkalemia, limitation of excess potassium load and cooking strategies should be implemented, together with close monitoring of serum potassium levels [45]. However, it is noteworthy that the increased risk of hyperkalemia due to high potassium load on vegetarian diets can be counteracted by the better correction of metabolic acidosis and the increased intestinal transit due to vegetarian diet. In CKD patients, no changes in serum potassium was observed using a vegan or an animal-based low-protein diet [46]. Similarly, during high-fruit intake, no hyperkalaemia episodes were reported [22].

The capacity of the colon/rectum to secrete potassium is inversely related to the residual renal function, and it substantially contributes to potassium homeostasis in patients with CKD. A pharmacological approach to hyperkalemia is another effective approach to counterbalance potassium overload in CKD [47]. 

The adoption of a plant-based diet such as the vegan or the very low-protein diet has positive effects on the bowel status, reducing the microbial catabolites originated by protein intestinal fermentation. In particular, p-cresyl sulfate, indoxyl-sulfate and TMAO (Trimethylamine *N*-oxide, an organic compound in the class of amine oxides), are reduced by protein restriction [48,49,50]. Moreover, increasing the fiber content in a diet has a modulating effect on gut microbiota composition, reducing protein fermentative processes and proteolytic metabolites [19,20]. 

The effects of diet on vitamin K status has been investigated for many years [51], but with non-definitive results. It should be always kept in mind that vitamin K status is dependent not only through dietary intake, but also intestinal bacterial synthesis, the endogenous recycling mechanisms and the interference of drugs, particularly warfarin, on the vitamin K cycle [52]. In addition, studies investigating dietary content of vitamin K should consider both phylloquinone (vitamin K1) and menaquinones (vitamin K2) content and only some of the available studies did so. 

Vitamin K has been favorably associated with various health outcomes, including diabetes [53], non-fatal cardiovascular diseases [54,55] and cancer [56]. The association between vitamin K intake and mortality has also been investigated. In a Dutch cohort of 33,289 participants with normal renal function, aged 20–70 years at baseline with a mean follow-up of 16.8 years, phylloquinone and menaquinones dietary intake was assessed with a food frequency questionnaire: vitamin K intake was not associated with all-cause mortality, cancer mortality and mortality from other causes [57]. On the other hand, an increase in dietary intake of vitamin K was found to be associated with a reduced risk of cardiovascular disease, cancer, or all-cause mortality in a Mediterranean population at high cardiovascular disease risk [58]. Similarly, vitamin K intake was associated with reduced mortality in people with chronic kidney disease [59]. 

Vitamin K is present in humans under different forms: vitamin K1 (phylloquinone, PK) and multiple K2 (menaquinones, MKs) vitamers which are classified according to the length of their unsaturated side chains. PK is found in green leafy vegetables. In particular, the best food sources of PK are cabbage kimchi, spinach, spring onions and soybean oils. Vitamin K2 is contained in cheese and fermented foods, such as natto (fermented soybean, common in Japan). Natto contains mainly MK-7, then MK-8 and PK in modest concentration. Usually, 75% to 90% of total vitamin K intake comes from PK, 10% to 25% derives from MKs. Importantly, MKs are also produced by intestinal bacterial fermentation. Considering that in the CKD population, a gut microbiota dysbiosis is present, a defective production of MKs may also occur [33].

Bacterial menaquinones present in the colon are mainly MK-10 and MK-11, synthesized by Bacteroides, MK-8 by Enterobacteria, MK-7 by Veillonella, and MK-6 by Eubacterium lentum [60]. Menaquinone-4 (MK-4) is the only one not derived by bacterial origin but by a direct conversion of dietary PKs [60].

PK content is quite high in all four renal diets considered here and largely covers the adequate intake of vitamin K for the general population that in Italy is set at 140 mcg/day between 30 and 59 years and 170 mcg/day over 60 years [29]. In 2001, the US Food and Nutrition Board suggested 120 mcg/day for adult males and 90 mcg/day for adult females [61]. The dietary PK content is strictly dependent on the variety of vegetables. In the planning of the daily meals of the four renal diets considered here the variability of vegetables was the same. The higher content of PK on VD and VLPD is due to a greater amount of vegetables. This is also the reason for the higher potassium load of VD and VLPD, although it does not exceed the WHO recommended minimum daily intake of 90 mmol/day (Table 4).

In the clinical setting, we found a remarkably low PK dietary intake in hemodialysis patients, 2-fold lower than the median intake suggested by RDAs for the Italian population [62]. An increase in vegetable foods as outlined in the VD could correct PK deficiency, both in CKD patients and in the general population, improving bone and vascular health. In particular, bone fractures are a common and remarkable complication in CKD patients [63]. In the large and diverse international DOPPS study, Tentori et al. [64] described a lower incidence of fractures in the Japanese population, where MKs dietary intake is high due to a large consumption of natto. Consistently, we found that PK deficiency was the strongest predictor of vertebral fractures in hemodialysis patients [65]. Prevalence of vertebral fractures was 55%, with a significant association between severity of fractures and lower levels of total BGP [66]. Currently, there is very little literature on the role of vitamin K supplementation in osteoporosis prevention in men and women. Osteoporosis, predisposing to an increased risk of fracture, is characterized by compromised bone strength, which is a result of two integrated main features, bone mineral density and bone quality. Although poor vitamin K status has been linked to osteoporosis in observational studies, no changes in bone mineral density were observed after PK supplementation [59]. However, Cheung et al. [67] described a lower incidence of clinical fractures among women treated with PK at the dose of 5 mg/day, despite a lack of effect on bone mineral density and reabsorption. Therefore, vitamin K could act on bone quality, in particular on microarchitecture or on mineralization patterns mediated by BGP action, rather than on bone mass density. In Japan, MK-4 is considered an effective drug for osteoporosis care, as it was shown to prevent fractures [68]. 

Although the MKs production by intestinal microbiota cannot be quantified, it might have a significant effect on bone and vascular health. Future studies should evaluate if diets rich in vegetables with prevalent intake of PK can influence the long chain MKs producing bacterial flora. Animal food rich in MKs could also contribute in improving the activity of vitamin K dependent proteins [69].

Neither the vegan diet nor a very low-protein diet include food from animal origin and they are therefore poor in vitamin B12 and in highly bio-available iron. These are the most important shortcomings of vegan/vegetarian diets. In the case of long-term treatment, preparations containing iron or vitamin B12 must be supplemented. Another potential shortcoming may be related to a potential lack of essential amino acids. However, a careful combination of pulses and cereals may give an adequate essential amino acids supply, enough to guarantee a positive nitrogen balance [46]; in the case of the very low-protein diet, supplementation of essential amino acids and ketoacids is mandatory to maintain a good nutritional status [70]. In addition, the exclusion of food of animal origin could determine a zinc deficiency. Zinc content in food of plant origin is lower than that of food of animal origin and scarcely absorbable do to the presence of fibers and phytate. Recently, it has been reported that zinc deficiency is seen in CKD patients and that heavy metal deficiency including zinc deficiency is one of the causes of Erythropoietin-resistant anemia [71]. Moreover, zinc deficiency is known to affect taste that is one of the most important factors in food choice, selection and consumption and it can affect appetite leading to dietary restrictions that could negatively impact nutritional and health status [72]. Hence, iron status, VitB12 zinc levels should be monitored in CKD patients in general and in particular in the elderly and in those following a vegan or very low protein diet for a long time. 

## 5. Conclusions

In conclusion, renal diets for advanced chronic renal insufficiency are structured to achieve the main goal of lowering protein, phosphate and sodium intake, while supplying adequate energy. The aim is to prevent or correct signs, symptoms and complications of renal insufficiency, delaying the start of dialysis and preserving nutritional status. Furthermore, renal diets may provide additional benefits, and this is the case for vegetarian diets. Namely, VD and VLPD also provide high amounts of fiber and Vitamin K1, with a very low acid load. These features may have favorable effects on Vitamin K status, intestinal microbiota and acid-base balance. Hence, we can speculate potential beneficial effects on vascular calcification and bone disease, on colonic environment and on protein metabolism. In the case of vegetarian diets, attention must be paid to serum potassium levels. 

## Figures and Tables

**Table 1:** General characteristics of the four renal diets (Ref. [23]), according to quantity and source of protein content.

	Normal Diet	Low Protein Diet	Vegan Diet	Very Low Protein Diet
Grains (bread, pasta, rice, barley, etc.)	6–8 servings per day: e.g., 1 serving at breakfast, 2 servings at lunch and dinner, 1 serving at snack	Replaced by protein-free products. 6–8 servings per day: e.g., 1 serving at breakfast, 2 servings at lunch and dinner, 1 serving at snack	6–8 servings per day: e.g., 1 serving at breakfast, 2 servings at lunch and dinner, 1 serving at snack	Replaced by protein-free products. 1 serving per day of grains is allowed to give variety to the diet. The amount is defined to obtain a daily protein intake of 0.3 g protein/kg body weight
Vegetables and fruits *	4–5 servings per day with suggestion to control potassium level	4–5 servings per day with suggestion to control potassium level	4–5 servings per day with suggestion to control potassium level	More than 4–5 servings per day with suggestion to control potassium level
Meat and Poultry	1 serving 1–2 times per week in the amount defined by the dietitian	1 serving per day as they represent the only source of proteins with high biological value. The daily amount is defined according to ideal/adjusted body weight and clinic	Excluded	Excluded
Fish	1 serving 2–3 times per week in the amount defined by the dietitian	Used as an alternative to meat and poultry	Excluded	Excluded
Beans (beans, chickpeas, peas, lentils, etc.)	At least 3 servings per week as a substitute for meat, fish etc., together with grains and not as a side dish	1–2 servings per week use together with rice, corn or regular bread and pasta (not with protein free products)	1 serving per day. Mandatory at least in one meal	1 serving 2–3 times per week, the amount is defined to obtain a daily protein intake of 0.3 g protein/kg body weight
Dairy products	1 serving of soft cheese (i.e., Mozzarella or ricotta cheese) every 7–10 days; 1 serving per day of milk or yogurt is allowed according to patient’s clinic. Avoid hard cheese	Excluded	Excluded	Excluded
Eggs	1 whole egg every 7–10 days. 2 egg whites in place of 35 g of meat or 50 g of fish 1–2 times per week	2 egg whites in place of 35 g of meat or 50 g of fish	Excluded	Excluded
Oil and fats ^#^	Olive oil intake is preferable in respect to animal fats	Olive oil intake is preferable in respect to animal fats	Olive oil intake is preferable in respect to animal fats	Olive oil intake is preferable respect to animal fats

Jam, marmalade, honey, sugar are allowed to increase energy content with the exception for diabetes, overweight or obesity. Butter or cream are allowed in the case of poor appetite or to improve food palatability and energy intake Importantly, VLPD does not cover the necessary daily amount of essential amino-acids, inducing the need for a supplementation with specific products, namely amino-acids and keto-acids. * In the case of hyperkalemia: reduce the number of servings, select fruits, use boiling. ^#^ Reduce the number of servings in the case of overweight/obesity.

**Table 2:** Energy and nutrients content of the four studied renal diets.

	ND	LPD	VD	VLPD	*p*
Energy, Kcal	1995 ± 63	2234 ± 123 ^a^	2190 ± 88 ^b^	2166 ± 197 ^c^	<0.001
Protein, g	57.6 ± 2.4	42.4 ± 2.5 ^a^	45.9 ± 3.5 ^b,d,e^	20.0 ± 3.2 ^c,f^	<0.001
Animal protein, g	16.8 ± 7.4	26 ± 9.6 ^a^	0.9 ± 1.6 ^b,d^	0.6 ± 1.0 ^c,f^	<0.001
Vegetable protein, g	35.0 ± 6.4	11 ± 3.5 ^a^	45 ± 5.5 ^b,d,e^	19.1 ± 1.9 ^c,f^	<0.001
Total fat, g	72.5 ± 5.4	91 ± 13 ^a^	79 ± 9.8	84 ± 14	<0.01
Saturated fat, g	14.6 ± 3.7	19 ± 5.4	17.4 ± 6.6	17 ± 4.5	n.s.
Unsaturated fat, g	50.7 ± 3.5	55.6 ± 8.2	55.4 ± 8.7	52 ± 11	n.s.
Cholesterol, mg	105 ± 83	107 ± 21	34 ± 39 ^b,d^	38 ± 29 ^c,f^	<0.01
Total carbohydrates, g	297 ± 16.4	331 ± 40	341 ± 23	351 ± 41	<0.01
Sugars, g	93.0 ± 11.4	84 ± 18	96 ± 11	104 ± 10 ^f^	<0.01
Starch, g	194 ± 11	172 ± 36	235 ± 18 ^b,d,e^	176 ± 50	<0.001
Fiber, g	28 ± 4.6	17 ± 3.4	35 ± 4.2 ^b,d,e^	25 ± 4.6 f	<0.001
Sodium, mg	649 ± 185	410 ± 229	647 ± 653	303 ± 218	n.s.
Potassium, mg	2265 ± 406	1969 ± 268	3152 ± 685 ^b,d^	2590 ± 645 ^f^	<0.001
Calcium, mg	476 ± 136	192 ± 53 ^a^	334 ± 73 ^b^	317 ± 74 ^c,f^	<0.001
Phosphorus, mg	884 ± 85	531 ± 72 ^a^	745 ± 56 ^b,d,e^	428 ± 87 ^c,f^	<0.001
Magnesium, mg	179.5 ± 37.1	109.5 ± 31.8	248.5 ± 56.9 ^b,d,e^	160.6 ± 72.1	<0.001
Iron, mg	9.60 ± 1.44	5.91 ± 1.23 ^a^	10.5 ± 1.65 ^c,d,f^	7.15 ± 2.20	<0.01
Copper	2.18 ± 1.45	1.10 ± 0.52	1.68 ± 0.93	1.55 ± 1.15	n.s.
Zinc, mg	6.40 ± 0.50	5.46 ± 1.38	5.36 ± 0.87	3.31 ± 1.11 ^c,e,f^	<0.001
Vitamin K1, μg	381.3 ± 220.2	185.7 ± 151.2	447.3 ± 410.0	441.6 ± 463.8	n.s.
Vitamin A, μg	590.3 ± 232.5	562.2 ± 384.5	951.4 ± 829.4	1214 ± 970.1	n.s.
Vitamin D, μg	0.52 ± 0.48	0.71 ± 0.66	0.51 ± 0.66	0.22 ± 0.27	n.s.
Vitamin B_12_, μg	1.51 ± 0.80	2.16 ± 1.42	0.03 ± 0.05 ^b,d^	0.02 ± 0.03 ^c,f^	<0.001

ND: normal diet (0.8 g protein/kg body weight); LPD: low protein diet (0.6 g protein/kg body weight); VD: vegan diet (0.7 g protein/kg body weight); VLPD: very low protein diet (0.3 g protein/kg body weight). Superscript letters indicate *p* < 0.05. ^a^: LPD vs. ND; ^b^: VD vs. ND; ^c^: VLPD vs. ND; ^d^: VD vs. LPD; ^e^: VLPD vs. VD; ^f^: VLPD vs. LPD. n.s.: not significant.

**Table 3:** Fiber, potassium, magnesium and Vitamin K1 content of the dietary patterns for chronic kidney disease (CKD) patients, as normalized per 1000 Kcal.

	ND	LPD	VD	VLPD	*p*
Fiber, g/1000 Kcal	10.4 ± 2.27	7.66 ± 1.60 ^a^	15.9 ± 2.21 ^d,e^	11.4 ± 2.15 ^c,f^	0.001
Vitamin K1, μg/1000 Kcal	190.8 ± 108.9	82.3 ± 65.6	206.5 ± 192.2	200.6 ± 203.1	n.s.
Potassium, mg/1000 Kcal	1135 ± 201.2	884.1 ± 132.8	1440 ± 306.3 ^b,d^	1200 ± 294.1 ^f^	0.001
Magnesium, mg/1000 Kcal	90.0 ± 18.9	49.2 ± 14.6 ^a^	113.4 ± 25.5 ^d,e^	74.5 ± 32.9	0.001

ND: normal diet (0.8 g protein/kg body weight); LPD: low protein diet (0.6 g protein/kg body weight); VD: vegan diet (0.7 g protein/kg body weight); VLPD: very low protein diet (0.3 g protein/kg body weight). Superscript letters indicate *p* < 0.05. ^a^: LPD vs. ND; ^b^: VD vs. ND; ^c^: VLPD vs. ND; ^d^: VD vs. LPD; ^e^: VLPD vs. VD; ^f^: VLPD vs. LPD.

**Table 4:** Recommended levels of nutrients intake of fiber, Vitamin K1, potassium and magnesium, for the general population in Italy (Livelli di Assunzione Raccomandata di Nutrienti, LARN) (ref. no. [29]) and by the National Kidney Foundation (NKF) (ref. no. [30]).

	LARN	NKF		
	General Population	CKD (stage 1–5 ND)	HD	PD
Fiber	12.6–16.7 g/1000 kcal	20–30 g/day	20–25 g/day	20–25 g/day
Vitamin K	30–59 years : 140 μg/day >60 years: 170 μg/day	90–120 μg/day (10 mg/day with antibiotic therapy)		
Potassium	3.9 g/day	Unrestricted unless serum level is high	Up to 2.7–3.1 g/day; adjust to serum levels	3–4 g/day; adjust to serum levels
Magnesium	240 mg/day	Not available	200–300 mg/day	Not available
